# Hydrodynamic Modeling and Parameter Identification of a Bionic Underwater Vehicle: RobDact

**DOI:** 10.34133/2022/9806328

**Published:** 2022-05-31

**Authors:** Qiyuan Cao, Rui Wang, Tiandong Zhang, Yu Wang, Shuo Wang

**Affiliations:** ^1^State Key Laboratory of Management and Control for Complex Systems, Institute of Automation, Chinese Academy of Sciences, Beijing, China; ^2^School of Artificial Intelligence, University of Chinese Academy of Sciences, Beijing, China; ^3^Center for Excellence in Brain Science and Intelligence Technology, Chinese Academy of Sciences, Shanghai, China

## Abstract

In this paper, the hydrodynamic modeling and parameter identification of the RobDact, a bionic underwater vehicle inspired by Dactylopteridae, are carried out based on computational fluid dynamics (CFD) and force measurement experiment. Firstly, the paper briefly describes the RobDact, then establishes the kinematics model and rigid body dynamics model of the RobDact according to the hydrodynamic force and moment equations. Through CFD simulations, the hydrodynamic force of the RobDact at different speeds is obtained, and then, the hydrodynamic model parameters are identified. Furthermore, the measurement platform is developed to obtain the relationship between the thrust generated by the RobDact and the input fluctuation parameters. Finally, by combining the rigid body dynamics model and the fin thrust mapping model, the hydrodynamic model of the RobDact at different motion states is constructed.

## 1. Introduction

The design of robot continues to garner considerable research attention for the application in different working environments. Xu et al. developed independent control strategy of multiple magnetic flexible millirobots for position control and path following [1, 2]. Wang et al. reviewed the system development and motion control of the bionic underwater robots [3]. Namiki and Yokosawa developed a double manipulator system, which can imitate human beings to complete the complex manual origami operation [4]. So et al. designed a flexible manipulator deformation sensor based on elastic sensor, which can be used to assist the manipulator to obtain tactile and other complex information [5].

At present, most of the traditional underwater robots are driven by a propeller, which is effective to cruise in the wide sea area at a stable speed. However, the underwater robot often needs to move or hover at a low speed with turbulence when performing a specific task, which makes the propeller difficult to meet the actual working condition [6]. In addition, due to the “twitching” movement of the propeller in the unstable flow, unpredictable fluid pulses will be generated and its efficiency will be significantly reduced. The bionic underwater robots have been widely utilized as valid tools on many marine tasks [7], including fishery industry, underwater life exploration, simultaneous localization and mapping, and fluid mapping [8–10]. In nature, fish swim with their central/pectoral fins at a slow speed to achieve more stability. In the case of high-speed swimming, relying on body/caudal fin propulsion can produce stronger thrust [11, 12]. In complex environment, compared with traditional underwater propulsion vehicle, bionic underwater vehicle has stronger hydrodynamic efficiency, robustness, and operability [13–15]. Besides, bionic underwater robots produce tiny noise and generate unattractive wake which make it environmental friendly.

In recent years, universities and research institutions around the world have designed and manufactured a variety of bionic underwater vehicles. Curet et al. developed and manufactured a series of bionic black devil knife fish and then carried out a series of experiments to study on the undulating propulsion performance [16, 17]. Zhou and Low created a bionic vehicle based on manta ray by using symmetrical double fins [18]. Xie and Shen designed a bionic underwater vehicle which has four undulating fin, and experiments show that the cooperative work of multiple undulating fins can improve the motion stability of the bionic underwater vehicle [19]. Xu et al. developed a helical microrobot driven by magnetic fields and proposed some strategies on path following and millirobot position control [20, 21]. Experiments show that the soft microrobot has obtained the excellent movement skills. Our laboratory is also committed to the research of underwater vehicle in this field. Wang et al. designed and manufactured a bionic underwater vehicle with symmetrical double undulatory fins [22] and carried out a series of research on path generation, path tracking control, and so on [23–25]. Zhang et al. designed a bionic underwater vehicle imitating the Dactylopteridae and studied the position control based on reinforcement learning [26, 27].

Underwater robot will be affected by the surrounding fluid when moving in the water, this phenomenon is called hydrodynamic effect, and a precise hydrodynamic model is needed. Specifically, the underwater vehicle must deal with unknown water flow and force, which may lead to unnecessary changes in position and attitude [25]. Bionic underwater vehicle is a multivariable, hysteretic, and nonsmooth system with many physical constraints. The modeling of it is usually very complex and difficult. In addition, the real underwater environment is changeable and difficult to predict, and the model parameters may shift with the change of the environment. Hydrodynamic model of underwater vehicle has strong nonlinearity and uncertainty, and there is still no unified modeling method at present [28]. With the improvement of hydrodynamics theory and the development of computer technology, the method of obtaining hydrodynamic coefficient through computational fluid dynamics (CFD) method has been widely extension. Chen et.al present a hydrodynamic model, which is proposed for a biomimetic robotic fish propelled by an ionic polymer-metal composite actuator [29]. The model incorporates both IPMC actuation dynamics and the hydrodynamics and predicts the speed of the robot under a given periodic actuation voltage. Kopman and Porfiri present a bionic robotic fish with a modular caudal fin and analyze its performance [30]. Liu et al. developed an underwater bionic robot and built a hydrodynamic model for undulating fin by the CFD method [31], which provides a rationale basis for investigating the effect of fluid flow field around the mechanical fin on propulsive performance. Ma et al. obtained the data-driven control strategy of the undulated fin underwater vehicle through experiments [32]. Bai et al. study the hydrodynamic effects of the different types of flippers by using the CFD method [33]. Bai et al. obtained the hydrodynamic coefficients of a long endurance underwater vehicle through the CFD simulation, and the hydrodynamic model is established according to the hydrodynamic coefficients [34]. However, it is difficult to connect the hydrodynamic force generated by underwater vehicles with the thrust generated by themselves, which makes the hydrodynamic model less applicable. We combine the CFD simulation and experiment to accurately construct the hydrodynamic model of the RobDact, which lays a foundation for the subsequent control research of the RobDact.

In this paper, hydrodynamic modeling and parameter identification of the RobDact are developed. Firstly, we briefly introduce the mechanical structure and motion modes of the RobDact, which is a Dactylopteridae-inspired biomimetic underwater vehicle, then establish the kinematics model and rigid body dynamics model of the RobDact, and give the hydrodynamic force equations based on the above model. The hydrodynamic force of the RobDact at different speeds is determined by CFD simulation. Furthermore, a measurement platform is developed that can measure the force of underwater vehicle in the *X*, *Y*, and *Z* direction. We establish a mapping relationship between the RobDact fluctuation parameters and the thrust by using force measurement experiments. Finally, by merging the rigid body dynamic model with the thrust mapping model, we develop a hydrodynamic model of the RobDact in various motion modes.

## 2. Rigid Body Dynamic Model

### 2.1. A Brief Introduction of the RobDact

**Figure 1:**
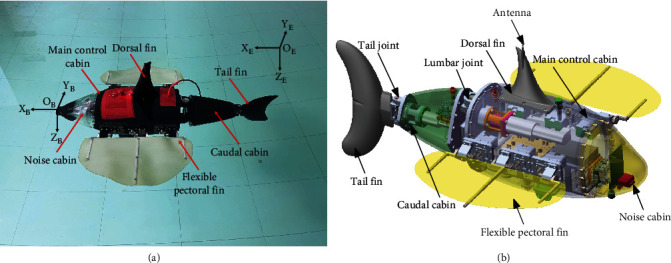
System configuration of the RobDact: (a) prototype; (b) 3D model.

The mechanical structure of the RobDact [26] is depicted in Figure 1. RobDact is composed of three compartments and three bionic fins: a noise cabin, a main control cabin, and a caudal cabin. Each pectoral fin is an undulating fin comprised of three fin rays that are separately actuated by the steering gear, allowing the RobDact to attain increased low-speed stability. The main control cabin is connected to the caudal cabin by a lumbar joint and is powered by a 200 W direct current motor. The caudal fin is connected to the caudal cabin through joints and is powered by a 90 W direct current motor. This results in the formation of a tail with multiple joints, which considerably improves the mobility of the RobDact.

### 2.2. Kinematic Model of the RobDact

**Figure 2:**
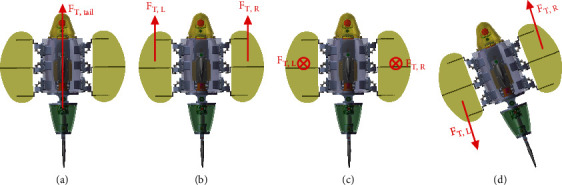
Four modes of motion: (a) high speed forward; (b) low speed forward; (c) snorkeling; (d) yaw.

RobDact has four motion modes; they are low speed forward, high speed forward, yaw, and snorkeling. The pectoral fin and caudal fin swing differently in different motion modes, as illustrated in Figure 2.

- Low speed forward. When the RobDact going forward or making a turn at slow speed, it generates propulsion by using the pectoral fin. The propulsion generated by the RobDact may be modified by adjusting the frequency and amplitude of the traveling wave transmission through the undulating fins on both sides. In this situation, the single undulating fin motion waveform can be considered as sinusoidal wave, and the motion of a single fin can be defined as

(1) $$\begin{array}{c} \theta \left(t\right)=\Theta \sin2\pi \left(\text{ft}+\phi \right)+\psi , \end{array}$$

where Θ is the maximum motion angle of the fin, *f* is the motion frequency of the fin, 2*πϕ* is the beginning phase of the fin, and *ψ* is the initial location of the fin

(ii) Snorkeling. While snorkeling, the RobDact obtains buoyancy mostly through the flapping of the pectoral fins. At this point, both sides of the pectoral fins are swinging at the same frequency, and the phase difference between the fins is zero. By adjusting the angle between the pectoral fin and the horizontal plane of the body, the amplitude and direction of the force generated by the undulating fins on both sides may be altered. At this point, one can represent the motion of a single undulating fin as

(2) $$\begin{array}{c} \theta \left(t\right)=\Theta \sin2\pi ft \end{array}$$

(iii) High speed forward. When the RobDact is going forward at high speed, it obtains thrust primarily through the cooperative swing of the caudal cabin and caudal fin. At the moment, the motion of caudal cabin and caudal fin can be defined as follows:

(3) $$\begin{array}{c} \left\{\begin{array}{c} \theta_{1}\left(t\right)=\Theta_{1}\sin2\pi \left(\text{ft}+\phi_{1}\right),\\ \theta_{2}\left(t\right)=\Theta_{2}\sin2\pi \left(\text{ft}+\phi_{2}\right) \end{array}\right. \end{array}$$

(iv) Yaw. When the RobDact is yawing, similar to low speed forwarding, it also generates thrust via the pectoral undulating fins. The distinction is that yaw can be achieved by reversing the transmission direction of one-side undulating fin, such that the transmission direction of both-side undulating fins is opposite, as illustrated in Figure 2

### 2.3. Dynamic Model of the RobDact

The ratio of the distance from the center of gravity to the center of buoyancy to the height of the RobDact is 0.183. Since the RobDact has good stability due to large metacentric height, it is reasonable to neglect the motion in pitch and roll. As a result, this paper establishes the fixed coordinate system and the following coordinate system, as seen in Figure 1. The origin of the following coordinate system is fixed at the geometric center of the RobDact.

In the fixed coordinate system *E* − *xyz*, the posture of the vehicle can be represented by vectors:

(4) $$\begin{array}{c} \eta ={\left[x,y,z,\psi \right]}^{T}, \end{array}$$

where *x*, *y*, *z* is the distance along the three coordinate axes and *ψ* is the rotation angle in the horizontal plane of the vehicle.

In the following coordinate *O* − *xyz*, the velocity vector of the RobDact is

(5) $$\begin{array}{c} v={\left[u,v,w,r\right]}^{T}, \end{array}$$

where *u*, *v*, *w* is the linear velocity of the RobDact and *r* is the angular velocity of the RobDact on the coordinate axis.

Therefore, the coordinate transformation matrix (from *O* − *xyz* transformation to *E* − *xyz*) can be defined as follows:

(6) $$\begin{array}{c} J=\left[\begin{array}{ccc} \cos\left(\psi \right) & -\sin\left(\psi \right) & 0\\ \sin\left(\psi \right) & \cos\left(\psi \right) & 0\\ 0 & 0 & 1 \end{array}\right]. \end{array}$$

The force and moment vectors of the RobDact can be defined as

(7) $$\begin{array}{c} \tau ={\left[X,Y,Z,R\right]}^{T}. \end{array}$$

Based on the rule of conservation of momentum, the force balance equation of the RobDact can be stated as follows:

(8) $$\begin{array}{c} M_{RB}\dot{v}+C_{RB}\left(v\right)v=\tau , \end{array}$$

where *M*_*RB*_ is the inertia matrix and *C*_*RB*_(*v*) represents the Coriolis force and centripetal force matrices, which can be expressed as follows:

(9) $$\begin{array}{c} M_{RB}=\left[\begin{array}{cccc} m & 0 & 0 & -my_{g}\\ 0 & m & 0 & mx_{g}\\ 0 & 0 & m & 0\\ -my_{g} & mx_{g} & 0 & I_{z} \end{array}\right], \end{array}$$

(10) $$\begin{array}{c} C_{RB}\left(v\right)=\left[\begin{array}{cccc} 0 & 0 & 0 & -mv\\ 0 & 0 & 0 & mu\\ 0 & 0 & 0 & 0\\ mv & -mu & 0 & 0 \end{array}\right], \end{array}$$

where *m* denotes the mass of the RobDact, *x*_*g*_, *y*_*g*_ denotes the center of gravity, and *I* denotes the moment of inertia in the horizontal plane. The above parameters of the model are *m* = 17.446 kg, *x*_*g*_ = 0.0035 m, *y*_*g*_ = 0.0018 m, and *I*_*z*_ = 0.446 kg · m^2^. Therefore, it can be represented in the following form:

(11) $$\begin{array}{c} M_{RB}=\left[\begin{array}{cccc} 17.446 & 0 & 0 & -0.031\\ 0 & 17.446 & 0 & 0.061\\ 0 & 0 & 17.446 & 0\\ -0.031 & 0.061 & 0 & 0.446 \end{array}\right]. \end{array}$$

There are three parts of the force acting on the vehicle:

(12) $$\begin{array}{c} \tau =\tau_{\text{P}}+\tau_{\text{H}}+\tau_{\text{E}}, \end{array}$$

where *τ*_P_ is the propulsion force and torque generated by the vehicles themselves, *τ*_H_ is the force and torque generated by the hydrodynamic force, and *τ*_E_ is the environmental disturbance caused by other causes.

The thrust produced by the RobDact may be characterized as the thrust produced by three fundamental pectoral and caudal fin motions while the RobDact performs four fundamental motions: low speed propulsion, high speed propulsion, snorkeling, and yaw (pectoral fin sinusoidal propulsion, pectoral fin flapping, and caudal fin propulsion). The thrust produced *τ*_P_ can be stated as follows:

(13) $$\begin{array}{c} \left\{\begin{array}{c} \tau_{\text{P}}={\left[F_{\text{PT}},0,0,0\right]}^{T}\left(\text{low speed}\right),\\ \tau_{\text{P}}={\left[F_{\text{CT}},0,0,0\right]}^{T}\left(\text{high speed}\right),\\ \tau_{\text{P}}={\left[0,0,2\cos\varphi \cdot F_{PL},0\right]}^{T}\left(\text{snorkeling}\right),\\ \tau_{\text{P}}={\left[0,0,0,F_{\text{PT}}\cdot l/2\right]}^{T}\left(\text{yaw}\right), \end{array}\right. \end{array}$$

where *F*_PT_ is the average force created by the pectoral fins; *F*_PL_ is the average lateral thrust produced by single pectoral fin flapping; *F*_CT_ is the thrust generated by the propulsion of caudal fin; *φ* is the angle formed by the pectoral fin of the RobDact with the horizontal plane; and *l* is the distance between two undulating fins of the RobDact.

## 3. Hydrodynamic Force and Moment Model

When the underwater vehicle is in motion, a portion of the energy is lost as surface ripples or hydrodynamic force. The following equation can be used to express hydrodynamic force and moment [35]:

(14) $$\begin{array}{c} \tau_{\text{H}}=-M_{A}\dot{v}-C_{A}\left(v\right)v-D\left(v\right)v-g\left(\eta \right), \end{array}$$

where *M*_*A*_ is the hydrodynamic added mass matrix of vehicle, *C*_*A*_(*v*) is the Coriolis and centripetal force matrix, *D*(*v*) is the linear damping matrix, and *g*(*η*) is the hydrodynamic restoring force, which is ignored in this paper due to the close proximity to gravity and buoyancy of the RobDact, and the motion amplitude is small in the two degrees of freedom of pitch and roll. *M*_*A*_, *C*_*A*_(*v*), and *D*(*v*) are denoted by the following:

(15) $$\begin{array}{c} M_{A}=-\left[\begin{array}{cccc} X_{\dot{u}} & X_{\dot{v}} & X_{\dot{w}} & X_{\dot{r}}\\ Y_{\dot{u}} & Y_{\dot{v}} & Y_{\dot{w}} & Y_{\dot{r}}\\ Z_{\dot{u}} & Z_{\dot{v}} & Z_{\dot{w}} & Z_{\dot{r}}\\ N_{\dot{u}} & N_{\dot{v}} & N_{\dot{w}} & N_{\dot{r}} \end{array}\right], \end{array}$$

(16) $$\begin{array}{c} \begin{array}{c} C_{A}\left(v\right)=-\left[\begin{array}{cccc} 0 & 0 & 0 & a_{2}\\ 0 & 0 & 0 & -a_{1}\\ 0 & 0 & 0 & 0\\ -a_{2} & a_{1} & 0 & 0 \end{array}\right]\\ a_{1}=X_{\dot{u}}u+X_{\dot{v}}v+X_{\dot{w}}w+X_{\dot{r}}r\\ a_{2}=X_{\dot{u}}u+Y_{\dot{v}}v+Y_{\dot{w}}w+Y_{\dot{r}}r \end{array}, \end{array}$$

(17) $$\begin{array}{c} D\left(v\right)=-\left[\begin{array}{cccc} X_{u} & X_{v} & X_{w} & X_{r}\\ Y_{u} & Y_{v} & Y_{w} & Y_{r}\\ Z_{u} & Z_{v} & Z_{w} & Z_{r}\\ N_{u} & N_{v} & N_{w} & N_{r} \end{array}\right], \end{array}$$

where *u*, *v*, *w* is the linear velocity of the RobDact and *r* is the angular velocity of the RobDact on the *Z*-axis. $X_{u},Y_{u},X_{\dot{u}},Y_{\dot{u}}$, etc. are the parameters to be identified.

According to Equation (8), Equation (12), and Equation (14), the 4-DOF dynamic equation of the RobDact can be expressed as

(18) $$\begin{array}{c} \begin{array}{c} M\dot{v}+C\left(v\right)v+D\left(v\right)v=\tau ,\\ M=M_{RB}+M_{A},\\ C\left(v\right)=C_{RB}\left(v\right)+C_{A}\left(v\right). \end{array} \end{array}$$

### 3.1. Identification of Hydrodynamic Model Parameters

Following the establishment of the dynamic equation for the RobDact, in order to acquire an accurate link between the motion state and force and torque, it is necessary to acquire the hydrodynamic force while the RobDact is in motion. As a result, we get the identification of hydrodynamic parameters. We primarily use the solid-liquid coupling module to simulate in order to get the hydrodynamic force of the RobDact in a certain motion state and, ultimately, to identify the hydrodynamic parameters.

Prior to identification, it is critical to define the RobDact's simulated motion state. Assuming that the RobDact is always performing irregular microoperations, the different irregular micromotions of the RobDact on four degrees of freedom are defined, such that the velocity and acceleration of each degree of freedom are unrelated. Motion that is spatially limited can be described as follows:

(19) $$\begin{array}{c} v=v_{0}+v_{p}\sin\left(2\pi f_{v1}t\right)\sin\left(2\pi f_{v2}t\right), \end{array}$$

where *v* denotes the four-dimensional velocity of the RobDact; *v*_0_ denotes a constant; *v*_*p*_ denotes the amplitude of disturbance; and *f*_*v*1_ and *f*_*v*2_ denote the frequency of disturbance.

**Figure 3:**
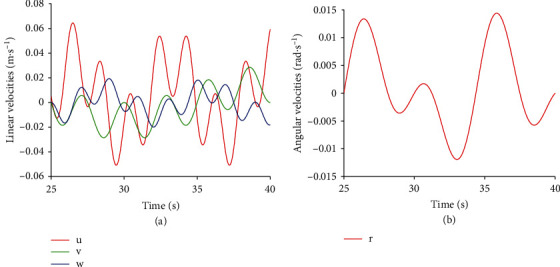
Simulation speed setting.

The 4-DOF motion of the RobDact is defined by Equation (19), with a step size of 0.05 s and a simulation time of 50 s.  Figure 3 depicts the linear and angular velocity of 25-40 s.

**Figure 4:**
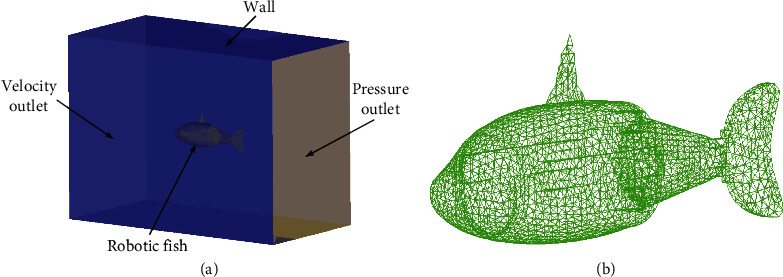
Simulation model and 3D mesh of the RobDact: (a) simulation model; (b) mesh.

Due to the tiny mass and volume of undulating fins, it is effective to ignore the hydrodynamic force generated by undulating fins. A simplified model of the RobDact with simply the main body is produced at a 1 : 1 ratio. As illustrated in Figure 4(a), an external fluid calculation domain and an internal rigid body calculation domain are generated. The external flow field is a cuboid domain with a size of 3*L*_*x*_ × 5*L*_*y*_ × 5*L*_*z*_ (*L*_*x*_, *L*_*y*_, and *L*_*z*_ equal to length, width, and height in *X*, *Y*, and *Z* directions of the RobDact). The outflow field's boundary condition is specified to be the velocity condition. To replicate the real-world swimming environment of the RobDact, the inlet water velocity is adjusted to 0.2 m/s, the normal swimming velocity of the RobDact in steady-state swimming under water. The pressure outlet is located at the outlet boundary. The opposite boundary of external flow field is referred to as the far field, as it lacks a sliding wall. After that, the RobDact and the flow field are meshed. Figure 4(b) depicts the mesh of the RobDact.

### 3.2. Hydrodynamic Parameter Identification

According to Equation (14), by using the multiple linear regression fitting method, hydrodynamic data obtained from CFD simulation is fitted to get the hydrodynamic parameters in Tables 1 and 2.

**Figure 5:**
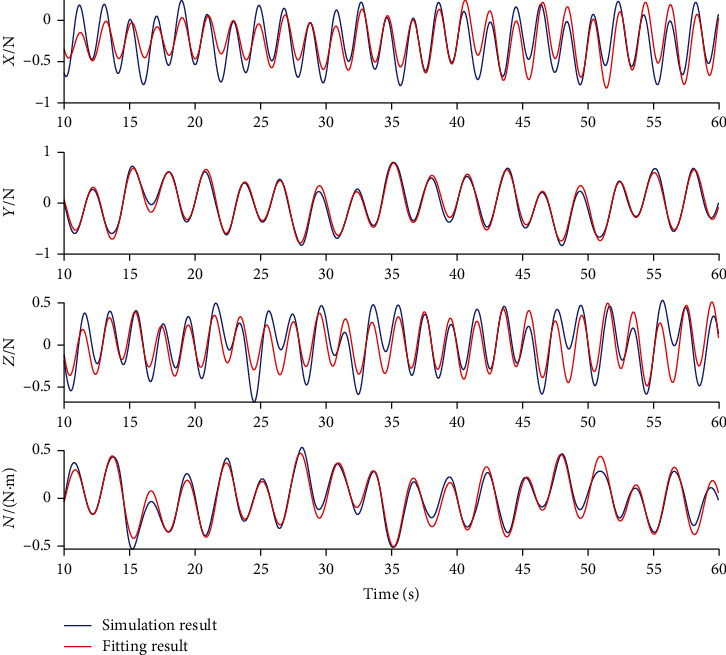
Comparison between CFD hydrodynamic simulation and fitting results.

Figure 5 shows the hydrodynamic force and torque results after parameter identification. As can be observed, the fitting results accord well with the CFD results, demonstrating that the hydrodynamic parameters of the regression model are accurate.

## 4. Fin Thrust Mapping Modeling

In the previous section, we establish a mapping link of the RobDact between motion state and hydrodynamic force. However, according to the kinematic model, it is important to construct a mapping model from fluctuation parameters (amplitude, frequency, and phase difference) to force. To begin, we discuss the measurement platform's structure, which is capable of measuring the force created by the underwater vehicle in three degrees of freedom (*X*, *Y*, and *Z*) in relation to various moving undulating characteristics. Then, we describe in detail how experimental strategy of the RobDact is designed during the measuring experiment.

### 4.1. Design of Measurement Platform

**Figure 6:**
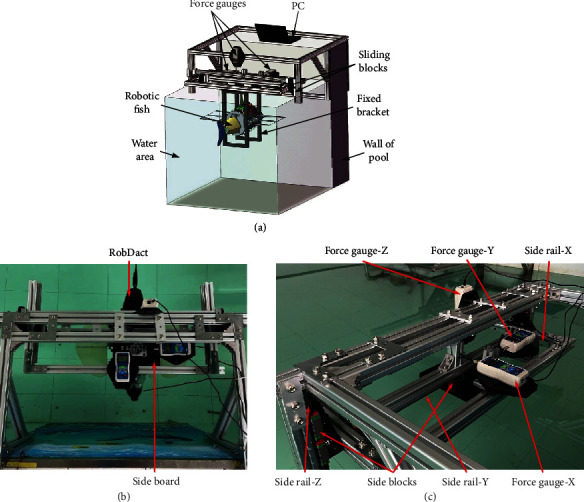
Schematic diagram of force measuring platform: (a) 3D model; (b) vertical view; (c) side view.

To determine the mapping relationship between the input fluctuation parameters and the generated thrust, a force measurement experiment must be conducted. The mechanical design of the force measurement platform described in this paper is shown in Figure 6. The device is attached to the wall of the pool, with the main body located within the water. The underwater vehicle is fixed with a bracket and completely submerged. The bracket is 3D printed, which is made of photosensitive resin and has a high degree of stiffness. When external force is applied on it, the deformation can be ignored and the force passed entirely to the acrylic sliding plate connected to the support above the water surface. Through the sliding block, the sliding plate is connected to the trapezoidal slide rail above. Due to the low overall friction of the sliding block, the effect of friction on the experiment may be ignored. To ensure steady functioning of the slider in each direction, two slide rails are fitted in each of the *X*, *Y*, and *Z* directions, and the force measuring platform has six slide rails. Each degree of freedom of *X*, *Y* and *Z* has a high-precision digital dynamometer for measuring the thrust component created by vehicle in three degrees of freedom. The dynamometer is attached to the aluminum profile support via an acrylic plate and has a measuring range of 500 N with an accuracy of 0.1 N. Each dynamometer is connected to a PC and is capable to read the value.

When the experiment begins, the instructions to the underwater vehicle are transmitted via PC and the goal fluctuation parameters are input to cause the vehicle to start moving in the target state and generate thrust. The supporter will hold the vehicle in place, and the generated force will be communicated to the dynamometer via the support, sliding plate, and sliding block. The force is finally conducted to the *X*, *Y*, and *Z* directions via the restriction by slide rail on the three degrees of freedom, allowing the dynamometer to measure the force on a single degree of freedom.

### 4.2. Experimental Configuration

As described above, the RobDact has four motion modes of motion. Without loss of generality, we divide three simplified motion modes: pectoral fin sinusoidal wave propulsion, pectoral fin snorkeling, and caudal fin propulsion. Following the acquisition of experimental data, the thrust generated by the RobDact in various motion modes can be computed.

To begin, the control parameters *v* = [*η*, *f*, Θ]^*T*^ of the RobDact are generated. When controlling an underwater vehicle, the primary parameters are the amplitude and frequency of the pectoral fin or caudal fin, where *f* is the input frequency, Θ is the input amplitude, and *η* is the sign of motion mode:

(20) $$\begin{array}{c} \eta =\left\{\begin{array}{c} 1\left(\text{pectoral fin sinusoidal wave propulsion}\right),\\ 2\left(\text{pectoral fin snorkeling}\right),\\ 3\left(\text{caudal fin propulsion}\right). \end{array}\right. \end{array}$$

The propulsive thrust also varies frequently throughout the movement of the RobDact due to the periodic movement of the fluctuation fin and caudal fin. To simplify the model, we focus on the average force generated by fin fluctuation. Create test data *d* and a vector of solution space *r* = [*F*_*x*_, *F*_*y*_, *F*_*z*_]^*T*^. Vector d represents the test data detected by the force measuring platform, and vector r represents the solution data in real enviroment, and *F*_*x*_, *F*_*y*_, *F*_*z*_ represent the average force in three directions.

(1) Pectoral fin sinusoidal waves. When the pectoral fin propels with a sinusoidal wave, the pectoral fins on both sides have the same amplitude and frequency, and the lateral pressures generated by the undulating fins on both sides are offset by one another. Due to the periodic motion of undulating fin, the average thrust in the vertical direction can be considered zero. The mobility modes can switch depending on the transmission direction of the traveling wave generated by the pectoral fins on both sides (if the traveling wave direction is positive, it will advance forward; if the traveling wave direction is positive and one pair, it will turn). Only the condition in which the traveling wave direction is identical to the positive direction is examined during the measurement experiment. To assist control, the phase difference is often set to 360/*n*, *n* = 1, 2, 3, ⋯, and in this paper, the phase difference is set to 60. Currently, the test data vector for pectoral fin propulsion is as follows:
  (21) $$\begin{array}{c} d={\left[T,0,0\right]}^{T}, \end{array}$$
  where *T* is the average force in the *X* direction detected by the measuring platform. The thrust produced by the RobDact can be thought of as a superposition of the thrust *d*_single_ produced by the individual undulating fins on both sides, i.e., *d*_single_ = *d*/2. At this point, because the caudal fin is not swinging, the overall attitude is fixed. The angle between the coordinate systems of the body and the world is zero (*ψ* = 0). As a result, the vector of the solution space *r* = *d*
The experimental input parameters *v*_pf_ during pectoral fin propulsion are
  (22) $$\begin{array}{c} v_{\text{pf}}={\left[\eta_{i},f_{i},\Theta_{k}\right]}^{T},\\ \eta_{\text{pf}}=1,\\ f_{i}=\left\{0.5\;0.6\;0.8\;1.0\;1.2\;1.4\;1.5\;1.6\;1.8\;2.0\;2.2\;2.4\right\}\left(\text{Hz}\right),\\ \Theta_{k}=10,20,30\left(\text{degrees}\right). \end{array}$$
(2) Pectoral fin snorkeling. Because the undulating fin does not move in the *X* direction during lateral flapping, the propulsive force is neglected at this point. Due to the periodic motion of undulating fin, the average vertical force can be considered to be zero. Therefore, the test space vector of pectoral fin propulsion is: *d* = [0, *L*, 0]^*T*^. *L* is the average amplitude of force in the *Y* direction detected by the measuring platform. At this point, because the caudal fin is not swinging, the overall attitude is fixed. The angle between the coordinate systems of the body and the world is zero (*ψ* = 0). As a result, the vector of the solution space *r* = *d*

During snorkeling, the following experimental parameters *v*_*s*_ are used:

(23) $$\begin{array}{c} v_{s}={\left[\eta_{i},f_{i},\Theta_{k}\right]}^{T},\\ \eta_{s}=2,\\ f_{i}=\left\{0.5\;0.6\;0.8\;1.0\;1.2\;1.4\;1.5\;1.6\;1.8\;2.0\right\}\left(\text{Hz}\right),\\ \Theta_{k}=10,20,30\left(\text{degrees}\right). \end{array}$$

**Figure 7:**
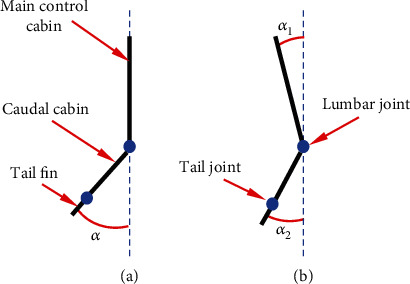
Caudal fin: (a) measurement platform; (b) real environment.

(3) Caudal fin propulsion. The force in the vertical direction is neglected during caudal fin propulsion, and the test data vector during caudal fin propulsion is as follows: *d* = [*T*_max_, *T*_min_, *L*, 0]^*T*^, where *T*_max_ is the maximum force in the *X* direction detected by the measuring platform, *T*_min_ is the minimize force in the *X* direction detected by the measuring platform, and *L* is the average amplitude of force in the *Y* direction. Because the angle between the tail and the head of the vehicle is continually shifting as the caudal fin swings. When determining the fixed coordinate system, coordinate transformation is required. It is set in the following coordinate system specified below. The included angle between the head of the RobDact and the *X*-axis of the fixed coordinate system is *α*_1_, and the angle between the tail and the *X*-axis of fixed coordinate system is *α*_2_. To simplify the model, the width of the RobDact is disregarded, as illustrated in Figure 7.

Due to the tiny mass and short length of caudal fin, the effect it caused on total swing angle of the RobDact is minimized. As a result, this paper treats the caudal fin and caudal cabin of the RobDact as a single unit, focusing exclusively on the *α*_1_ and *α*_2_. Therefore,

(24) $$\begin{array}{c} \alpha_{1}+\alpha_{2}=\alpha ,\\ \alpha =\Theta_{1}\sin2\pi \left(\text{ft}+\phi_{1}\right), \end{array}$$

where *α*s the swing amplitude of the tail in the following body coordinate system at the current time. According to the law of conservation of angular momentum,

(25) $$\begin{array}{c} J_{1}\alpha_{1}=J_{2}\alpha_{2},\\ \frac{\alpha_{1}}{\alpha_{2}}=\frac{J_{2}}{J_{1}}, \end{array}$$

where *J*_1_is the moment of inertia for the head of the RobDact and *J*_2_ is the moment of inertia for the tail of the RobDact. We can obtain *J*_1_ = 1.116 kg · m^2^ and *J*_2_ = 0.282 kg · m^2^.

Because of the sinusoidal swing of the tail, the force generated by the RobDact during caudal fin propulsion is periodic. The RobDact is symmetrical in the *X*-*Z* plane, so *F*_*y*_ = 0. The solution vector could be expressed as *r* = [*F*_*x*_, 0, 0]^*T*^, and *F*_*x*_ could be expressed as

(26) $$\begin{array}{c} F_{x}=\overset{¯}{F_{\text{forward}}}+\overset{¯}{F_{\text{lateral}}},\\ F_{\text{forward}}=\frac{T_{\max}+T_{\min}}{2}\sin\left(2\pi ft\right)\cos\left(\alpha_{1}\right),\\ F_{\text{lateral}}=\left|L\cos\left(2\pi ft\right)\sin\left(\alpha_{2}\right)\right|. \end{array}$$

During caudal fin propulsion, the following experimental parameters *v*_*s*_ are used:

(27) $$\begin{array}{c} v_{tf}={\left[\eta_{i},f_{i},\Theta_{k}\right]}^{T},\\ \eta_{tf}=3,\\ f_{i}=\left\{0.5\;0.6\;0.8\;1.0\;1.2\;1.4\;1.5\;1.6\;1.8\;2.0\right\}\left(\text{Hz}\right),\\ \Theta_{k}=10,20,30\left(\text{degrees}\right). \end{array}$$

## 5. Experimental Results and Discussion

In this section, we perform experiments under many kinds of dynamic fuzzy conditions and obtain experimental results. In Section 5.1, the appropriate formula of the experimental results was conducted. In Section 5.2, we get the parameters of fin thrust mapping model by identification of results. Then, by combining the fin thrust mapping model and hydrodynamic force and moment model, the complete hydrodynamic model of the RobDact is established.  Section 5.3 is a discussion section; the instructions of hydrodynamic model is introduced.

### 5.1. Fin Thrust Mapping Model

After receiving the experimental results, it is important to fit the data in order to derive the functional expression between the thrust and fluctuation parameters. To begin, an appropriate formula between the thrust and fluctuation parameters must be developed.

(1) When the RobDact propels and beats via the pectoral fin, this paper fits the data by using the polynomial formula, which may be created as follows:
  (28) $$\begin{array}{c} T_{p}\left(f,\Theta \right)=\overset{3}{\underset{n=1}{\sum }}\left[\left(a_{n}f^{3}+b_{n}f^{2}+c_{n}f+d_{n}\right)\left(e_{n}\Theta +f_{n}\right)\right], \end{array}$$
  (29) $$\begin{array}{c} L\left(f,\Theta \right)=\overset{3}{\underset{n=1}{\sum }}\left[\left(a_{n}f^{3}+b_{n}f^{2}+c_{n}f+d_{n}\right)\left(e_{n}\Theta +f_{n}\right)\right], \end{array}$$
  where *a*_*n*_, *b*_*n*_, *c*_*n*_, *d*_*n*_, *e*_*n*_, and *f*_*n*_ are the parameters to be identified and *f* and Θ are the operating frequency and amplitude, respectively; they are also the input undulating parameters of the RobDact
(2) When the RobDact propels through the caudal fin, if the link between thrust and frequency is fitted by using a polynomial formula, it may result in a poor precision. Thus, we develop fitting function by combining the exponential function and the polynomial formula, describe the relationship between thrust and frequency using the exponential function, and describe the change in thrust with amplitude using the quadratic term formula. The formula can be written as follows:
  (30) $$\begin{array}{c} T_{t}\left(f,\Theta \right)=\left(\overset{3}{\underset{n=1}{\sum }}a_{n}e^{-b_{n}{\left(f-c_{n}\right)}^{2}}\right)\times \left(d_{1}\Theta^{2}+d_{2}\Theta +d_{3}\right), \end{array}$$
  where *a*_*n*_, *b*_*n*_, *c*_*n*_, and *d*_*n*_ are the parameters to be detected and *f* and Θ are the frequency and amplitude of caudal fin swing, which are the RobDact input fluctuation parameters

### 5.2. Hydrodynamic Modeling Result

The functional equation between thrust, frequency, and amplitude generated by the RobDact can be fitted by using the curve fitting method. The functional relationship between thrust and fluctuation parameters in the pectoral fin sinusoidal waves and pectoral fin snorkeling modes can be expressed using Equation (28), and the parameters are listed in Tables 3 and 4.

The functional relationship between the thrust and fluctuation parameters in the caudal fin propulsion mode can be described using Equation (30), and the parameters are listed in Table 5.

**Figure 8:**
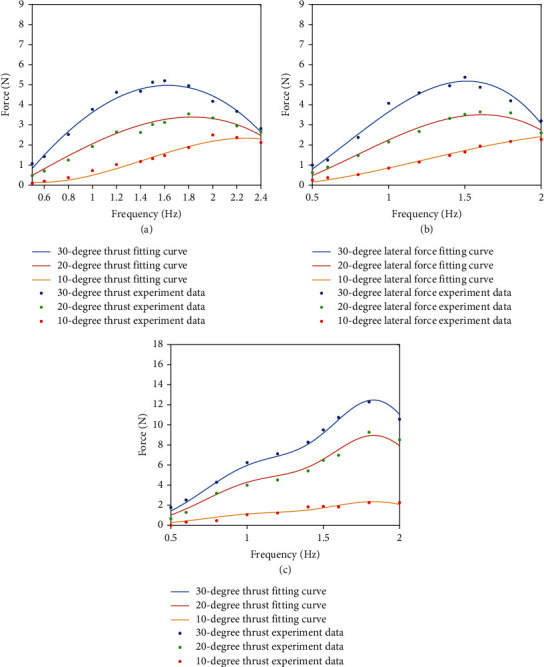
Fitting results compared with experimental data: (a) pectoral fin propulsion; (b) pectoral fin side beat; (c) caudal fin propulsion.

Comparing the fitting results with the experimental results, the results shown in Figure 8 are obtained. It can be seen from the picture, among three propulsion modes, when the amplitude of fins is fixed, as the frequency of the RobDact increases within limits, the force generated by the RobDact rises. After the frequency grows over the threshold value, the reason for frequency threshold that appears is the upper bound of the steering engine's performance. When the frequency is too high, the steering engine will be overburdened. As for 10° experiment, although there is no obvious threshold, the force will stop growing when the frequency is too high. The force begins to decrease sharply. So it is efficient to input the control parameters under the threshold value.

The process of developing hydrodynamic model of the RobDact can be separated into three stages: thrust calculation, hydrodynamic calculation, and motion state calculation.

(1) Calculation of thrust. When developing the RobDact hydrodynamic model, the thrust created by the model must be estimated in relation to motion mode. To begin, enter the traveling undulating parameters for the RobDact and put them into the fitting algorithms Equation (28) and Equation (30). The parameters of fitting formula are presented in Tables 3–5, which are used to compute the thrust generated by the fundamental movement of fin at this time. The thrust created by the fundamental motion of fin is then inserted into Equation (13) to determine the thrust *τ*_p_ generated by the RobDact as a whole in this motion state
(2) Calculation of hydrodynamics force. The hydrodynamic force *τ*_h_ exerted by the RobDact may be estimated using Equation (14). The additional mass matrix *M*_*A*_ and linear damping matrix *D*(*v*) parameters used in the formula are listed in Tables 1 and 2. The Coriolis force and centripetal force matrix *C*_*A*_(*v*) can be derived using Equation (14)
(3) Calculation of the motion state. To determine the state of motion of the RobDact, the combined external force and acceleration must be calculated. At this point, the external force *F*_*r*_ and acceleration $\dot{v}$ combined with the RobDact can be described as follows:
  (31) $$\begin{array}{c} F_{r}=\tau_{P}+\tau_{H}-C\left(v\right)v+D\left(v\right)v,\\ \dot{v}=M^{-1}\left[\tau_{P}+\tau_{H}-C\left(v\right)v+D\left(v\right)v\right], \end{array}$$
  where *C*(*v*) = *C*_*RB*_(*v*) + *C*_*A*_(*v*) is the matrix of Coriolis and centripetal forces, and the internal value *C*_*RB*_(*v*) and *C*_*A*_(*v*) can be computed using Equitation (10) and Equation (16); *M* is the mass matrix, and the value is as follows:
  (32) $$\begin{array}{c} M=\left[\begin{array}{cc} \begin{array}{ccc} 20.6222 & -0.0279 & -2.3930 \end{array} & 2.4674\\ \begin{array}{ccc} 0.1103 & 3.5012 & -0.2367 \end{array} & -12.8056\\ \begin{array}{ccc} -0.3547 & -0.0268 & 28.1553 \end{array} & -0.1749\\ \begin{array}{ccc} -0.1222 & 8.2168 & 0.0845 \end{array} & 9.2725 \end{array}\right]. \end{array}$$

### 5.3. Discussion

As shown in Figure 8, at the same frequency and amplitude, caudal fin propulsion mode produces more thrust than pectoral fin propulsion mode. So when we control the RobDact, it is more efficient to use the caudal fin propulsion mode at high speed and pectoral fin propulsion mode at low speed. It is very hard to control the underwater vehicles because there are many conditions to consider, such as the unknown water flow and disturbance generated by the vehicle. After we get the hydrodynamic model of the RobDact, the motion state of the RobDact could be calculated easily.

The calculation of the motion state is decomposed into four parts. First, we need to clarify the target force of the RobDact. Second, by using the hydrodynamic model, we get the hydrodynamic force and moment. Third, combining the target force and moment generated by the RobDact with hydrodynamic force and moment, the resulting force of the RobDact will be calculated. Finally, we could get the input parameters from the fin thrust mapping model.

## 6. Conclusions

The hydrodynamic modeling and parameter identification of the RobDact, a bionic underwater vehicle inspired by Dactylopteridae, have been performed based on CFD. To begin, this article introduces the study object RobDact, a Dactylopteridae-inspired biomimetic underwater vehicle, and establishes kinematics and rigid body dynamics models by using hydrodynamic and moment equations. CFD is used to simulate the hydrodynamic forces created by the RobDact at various speeds and subsequently to identify the hydrodynamic model parameters. Following that, we develop an experimental platform for force measurement and conduct experiments to determine fin thrust mapping relationship model of the RobDact. Finally, by combining the rigid body dynamics model and the fin thrust mapping relationship model, we develop a hydrodynamic model of the RobDact in various motion modes.

In future work, we intend to study the relationship between the underwater maximum swimming speed of RobDact and the thrust generated by the fin. Besides, experiments will be conducted to study the dynamic motion of the RobDact. Also, the experiments could be used to prove the validity of the assumptions we made in Section 4.2. Moreover, the intelligent control of bionic underwater vehicle will be conducted by using the RobDact hydrodynamic model in conjunction with some AI methods such as reinforcement learning.

## Figures and Tables

**Table 1:** Coefficient of hydrodynamic added mass matrix *M*_*A*_.

$X_{\dot{u}}$	-3.1762	$X_{\dot{v}}$	0.0279	$X_{\dot{w}}$	2.3930	$X_{\dot{r}}$	-2.4948
$Y_{\dot{u}}$	-0.1103	$Y_{\dot{v}}$	13.9448	$Y_{\dot{w}}$	0.2367	$Y_{\dot{r}}$	12.8666
$Z_{\dot{u}}$	0.3547	$Z_{\dot{v}}$	0.0268	$Z_{\dot{w}}$	-10.7093	$Z_{\dot{r}}$	0.1749
$N_{\dot{u}}$	0.0912	$N_{\dot{v}}$	-8.1558	$N_{\dot{w}}$	-0.0845	$N_{\dot{r}}$	-8.8265

**Table 2:** Coefficient of hydrodynamic linear damping matrix *D*(*v*).

*X*_*u*_	0.5996	*X*_*v*_	2.4967	*X*_*w*_	-7.2015	*X*_*r*_	-1.9539
*Y*_*u*_	-0.3623	*Y*_*v*_	11.0042	*Y*_*w*_	-0.9735	*Y*_*r*_	9.2471
*Z*_*u*_	1.6199	*Z*_*v*_	0.0229	*Z*_*w*_	6.1313	*Z*_*r*_	-0.8115
*N*_*u*_	0.2295	*N*_*v*_	-6.5004	*N*_*w*_	0.2103	*N*_*r*_	-7.3168

**Table 3:** Fitting parameters of pectoral fin sinusoidal wave.

*n*	1	2	3
*a*_*n*_	0.9262	0.1016	0.0535
*b*_*n*_	-1.6480	1.5580	0.7496
*c*_*n*_	1.5960	-2.0650	1.3190
*d*_*n*_	0.6419	1.4700	0.3941
*e*_*n*_	0.0442	-0.1958	0.1191
*f*_*n*_	-1.5290	3.0170	-0.7917

**Table 4:** Fitting parameters of pectoral fin snorkeling.

*n*	1	2	3
*a*_*n*_	0.2144	-0.0209	1.5570
*b*_*n*_	-0.0184	0.6404	-0.0029
*c*_*n*_	0.8122	0.2152	-1.3430
*d*_*n*_	0.2413	0.1511	1.2420
*e*_*n*_	-0.1166	0.3832	-0.0635
*f*_*n*_	-0.2066	-1.1090	0.5556

**Table 5:** Fitting parameters of caudal fin propulsion.

*n*	1	2	3
*a*_*n*_	0.0514	-1.7630	-0.8956
*b*_*n*_	-0.03102	2.2080	1.9130
*c*_*n*_	0.4961	1.8670	1.0820
*d*_*n*_	0.0085	-0.6171	4.0280

## Data Availability

The data used to support the findings of this study are available from the corresponding author upon request.

## References

[1] Xu T., Huang C., Lai Z., Wu X. (2022). Independent control strategy of multiple magnetic flexible millirobots for position control and path following. *IEEE Transactions on Robotics*.

[2] Xu T., Hao Z., Huang C., Yu J., Zhang L., Wu X. (2022). Multimodal locomotion control of needle-like microrobots assembled by ferromagnetic nanoparticles. *IEEE/ASME Transactions on Mechatronics*.

[3] Wang R., Wang S., Wang Y., Cheng L., Tan M. (2022). Development and motion control of biomimetic underwater robots: a survey. *IEEE Transactions on Systems, Man, and Cybernetics: Systems*.

[4] Namiki A., Yokosawa S. (2021). Origami folding by multifingered hands with motion primitives. *Cyborg and Bionic Systems*.

[5] So J., Kim U., Yong B. K., Seok D. Y., Choi H. R. (2021). Shape estimation of soft manipulator using stretchable sensor. *Cyborg and Bionic Systems*.

[6] Bandyopadhyay P. R. (2005). Trends in biorobotic autonomous undersea vehicles. *IEEE Journal of Oceanic Engineering*.

[7] Zheng J., Zhang T., Wang C., Xiong M., Xie G. (2022). Learning for attitude holding of a robotic fish: an end-to-end approach with sim-to-real transfer. *IEEE Transactions on Robotics*.

[8] Katzschmann R. K., DelPreto J., MacCurdy R., Rus D. (2018). Exploration of underwater life with an acoustically controlled soft robotic fish. *Science Robotics*.

[9] Mahon I., Williams S. B., Pizarro O., Johnson-Roberson M. (2008). Efficient view-based SLAM using visual loop closures. *IEEE Transactions on Robot*.

[10] Chang D., Wu W., Edwards C. R., Zhang F. (2017). Motion tomography: mapping flow fields using autonomous underwater vehicles. *International Journal of Robotics Research*.

[11] Wang Y., Wang R., Wang S., Tan M., Yu J. (2020). Underwater bioinspired propulsion: from inspection to manipulation. *IEEE Transactions on Industrial Electronics*.

[12] Kopman V., Laut J., Acquaviva F., Rizzo A., Porfiri M. (2015). Dynamic modeling of a robotic fish propelled by a compliant tail. *IEEE Journal of Oceanic Engineering*.

[13] Chu W. S., Lee K. T., Song S. H., Han M. W., Ahn S. H. (2012). Review of biomimetic underwater robots using smart actuators. *International Journal of Precision Engineering & Manufacturing*.

[14] Epstein M., Colgate J. E., Maciver M. A. Generating thrust with a biologically-inspired robotic ribbon fin.

[15] Elghazaly G., Gouttefarde M., Creuze V. Hybrid cable-thruster actuated underwater vehicle-manipulator systems: a study on force capabilities.

[16] Curet O. M., Patankar N. A., Lauder G. V., MacIver M. A. (2011). Mechanical properties of a bio-inspired robotic knifefish with an undulatory propulsor. *Bioinspiration & Biomimetics*.

[17] Curet O. M., Patankar N. A., Lauder G. V., MacIver M. A. (2011). Aquatic manoeuvering with counter-propagating waves: a novel locomotive strategy. *Journal of the Royal Society Interface*.

[18] Zhou C., Low K. H. (2012). Design and locomotion control of a biomimetic underwater vehicle with fin propulsion. *IEEE/ASME Transactions on Mechatronics*.

[19] Xie H., Shen L. Dynamic analysis on the bionic propulsor imitating undulating fin of aquatic animals.

[20] Xu S., Liu J., Yang C., Wu X., Xu T. (2021). A learning-based stable servo control strategy using broad learning system applied for microrobotic control. *IEEE Transactions on Cybernetics*.

[21] Xu T., Guan Y., Liu J., Wu X. (2020). Image-based visual servoing of helical microswimmers for planar path following. *IEEE Transactions on Automation Science and Engineering*.

[22] Wang R., Wang Y., Wang S., Tang C., Tan M. Modular design and control of an underwater biomimetic vehicle-manipulator system.

[23] Wang R., Wang S., Wang Y., Tan M., Yu J. (2019). A paradigm for path following control of a ribbon-fin propelled biomimetic underwater vehicle. *IEEE Transactions on Systems, Man, and Cybernetics: Systems*.

[24] Wang R., Wang S., Wang Y., Tang C. Path following for a biomimetic underwater vehicle based on ADRC.

[25] Wang R., Wang S., Wang Y., Cai M., Tan M. (2019). Vision-based autonomous hovering for the biomimetic underwater robot—RobCutt-II. *IEEE Transactions on Industrial Electronics*.

[26] Zhang T., Wang R., Wang Y., Cheng L., Wang S., Tan M. (2021). Design and locomotion control of a Dactylopteridae-inspired biomimetic underwater vehicle with hybrid propulsion. *IEEE Transactions on Automation Science and Engineering*.

[27] Zhang T., Wang R., Wang Y., Wang S. Locomotion control of a hybrid propulsion biomimetic underwater vehicle via deep reinforcement learning.

[28] Christ R. D., Wernli S. R. L. (2011). *The ROV Manual: A User Guide for Observation Class Remotely Operated Vehicles*.

[29] Chen Z., Shatara S., Tan X. (2010). Modeling of biomimetic robotic fish propelled by an ionic polymer-metal composite caudal fin. *IEEE/ASME Transactions on Mechatronics*.

[30] Kopman V., Porfiri M. (2013). Design, modeling, and characterization of a miniature robotic fish for research and education in biomimetics and bioinspiration. *IEEE/ASME Transactions on Mechatronics*.

[31] Liu F., Lee K.-M., Yang C.-J. (2012). Hydrodynamics of an undulating fin for a wave-like locomotion system design. *IEEE/ASME Transactions on Mechatronics*.

[32] Ma R., Du H., Wang R., Wang Y., Wang S., Chong T. Data-driven locomotive strategies of the UVMS propelled by undulating fins.

[33] Bai X., Wang Y., Wang R., Wang S., Tan M. (2022). Hydrodynamics of a flexible flipper for an underwater vehicle-manipulator system. *IEEE/ASME Transactions on Mechatronics*.

[34] Bai X., Wang Y., Wang S., Wang R., Tan M., Wei W. (2022). Modeling and analysis of an underwater biomimetic vehicle-manipulator system. *Science China Information Sciences*.

[35] Fossen T. I. (1994). *Guidance and Control of Ocean Vehicles*.

